# Does present use of cardiovascular medication reflect elevated cardiovascular risk scores estimated ten years ago? A population based longitudinal observational study

**DOI:** 10.1186/1471-2458-11-144

**Published:** 2011-03-02

**Authors:** Mette Brekke, Jørund Straand

**Affiliations:** 1 Department of General Practice/FamilyMedicine, Institute of Health and Society, University of Oslo, PO Box 1130 Blindern, 0318 Oslo, Norway; 2 Research Unit for General Practice, Institute of Health and Society, University of Oslo, PO Box 1130 Blindern, 0318 Oslo, Norway

## Abstract

**Background:**

It is desirable that those at highest risk of cardiovascular disease should have priority for preventive measures, eg. treatment with prescription drugs to modify their risk. We wanted to investigate to what extent present use of cardiovascular medication (CVM) correlates with cardiovascular risk estimated by three different risk scores (Framingham, SCORE and NORRISK) ten years ago.

**Methods:**

Prospective logitudinal observational study of 20 252 participants in The Hordaland Health Study born 1950-57, not using CVM in 1997-99. Prescription data obtained from The Norwegian Prescription Database in 2008.

**Results:**

26% of men and 22% of women aged 51-58 years had started to use some CVM during the previous decade. As a group, persons using CVM scored significantly higher on the risk algorithms Framingham, SCORE and NORRISK compared to those not treated. 16-20% of men and 20-22% of women with risk scores below the high-risk thresholds for the three risk scores were treated with CVM, while 60-65% of men and 25-45% of women with scores above the high-risk thresholds received no treatment. Among women using CVM, only 2.2% (NORRISK), 4.4% (SCORE) and 14.5% (Framingham) had risk scores above the high-risk values. Low education, poor self-reported general health, muscular pains, mental distress (in females only) and a family history of premature cardiovascular disease correlated with use of CVM. Elevated blood pressure was the single factor most strongly predictive of CVM treatment.

**Conclusion:**

Prescription of CVM to middle-aged individuals by large seems to occur independently of estimated total cardiovascular risk, and this applies especially to females.

## Background

The ability to predict future risk of cardiovascular disease (CVD) in asymptomatic persons, in order to implement preventive measures, is important for clinicians as well as for public health. Several risk predicting tools have been developed for this purpose [1-4] and electronic decision aids are available for rapid risk estimation in daily clinical work. Unfortunately, these tools are probably still underused in clinical practice, where interventions commonly have been based on assessment of single risk factors like blood pressure or serum cholesterol [5,6]. From a public health perspective it is desirable that those at highest risk should have priority for preventive measures, eg. with prescription drugs to modify their risk for CVD. It is, however, not known to which extent those at highest risk based on a total risk assessment correspond to those who during follow-up are actually treated with cardiovascular medication (CVM).

In this prospective longitudinal observational study in a Norwegian population born 1950-57, we investigated to which extent results from three different risk score algorithms (Framingham, SCORE and NORRISK) predicted use of CVM ten years later [1-3]. Our aim was to explore whether those being "at risk" according to their risk scores, corresponded to individuals who subsequently had been put on drug treatment. We also studied if factors normally not included in risk score algorithms-such as educational level, psychological distress, and self-evaluated general health-predicted use of CVM ten years later.

## Methods

### Study design, setting, participants, outcome variables, and data sources

In this population based, longitudinal observational study, a total of 20 252 men and women born 1950-57 and living in Hordaland County in Western Norway participated. Their individual cardiovascular risk was mapped in 1997-99, and in 2008 their use of CVM was investigated. Data sources were the Hordaland Health Study (1997-99) and the Norwegian Prescription Database (2008).

### The Hordaland Health Study (HUSK)

In 1997-99 a population based health survey was carried out in Hordaland County in Western Norway: The Hordaland Health Study (HUSK) [7]. Those invited were a randomly selected sample of men and women born in 1950 and 1951 (n = 4 849), and all inhabitants born between 1953 and 1957 (n = 29 400). The survey included a questionnaire, addressing smoking, medication use, self-reported diabetes mellitus and cardio- and cerebrovascular disease (including a family history). Participants also attended a mobile screening station provided by the National Health Screening Service, Oslo (now the Norwegian Institute of Public Health), where strictly standardized measurements of systolic-(SBP) and diastolic blood pressure (DBP), height, weight, cholesterol, triglycerides and glucosis levels were carried out. The population included in HUSK is found to be representative for the Norwegian population as such [7].

### Participants in the present study

Of the 33 549 individuals born 1950-57 invited to participate in HUSK, 22 289 (66.6%) responded: 10 249 men (59.1%) and 12 040 women (74.3%). The 9 283 men (90.6%) and 10 969 women (91.1%) not using any CVM in 1997-99, comprised the target population for the present study (Table 1).

**Table 1:** Age and sex distribution of participants (n = 22 298) in The Hordaland Health Study (HUSK) 1997-99 and participants (n = 20 252) included in the 2008 record linkage study, where individuals using cardiovascular medication (CVM) in 1997-99 had been excluded (n = 2 046)

*Birth year*	*1997-99*		*2008*	
	
	*Males n (%)*	*Females n (%)*	*Males n (%)*	*Females n (%)*
1950	881 (8.6)	1 067 (8.9)	833 (9.0)	1 028 (9.4)
1951	783 (7.6)	1 002 (8.3)	745 (8.0)	967 (8.8)
1953	1 725 (16.8)	1 982 (16.4)	1 661 (17.9)	1 755 (16.0)
1954	1 721 (16.8)	2 023 (16.8)	1 390 (15.0)	1 796 (16.4)
1955	1 703 (16.6)	2 036 (16.9)	1437 (15.5)	1 641 (15.0)
1956	1 721 (16.8)	1 996 (16.6)	1 647 (17.7)	1 931 (17.6)
1957	1 715 (16.7)	1 943 (16.1)	1 570 (16.9)	1 851 (16.9)

**Total**	10 249 (100%)	12 049 (100%)	9 283 (100%)	10 969 (100%)

### The Norwegian Prescription Database (NorPD)

The Norwegian Prescription Database (NorPD) provides information on all prescription drugs dispensed from Norwegian pharmacies [8]. The register includes information on patients' gender and date of birth, prescribers' age, sex and clinical specialty, and prescription details according to the Anatomical Therapeutic Chemical (ATC) classification system [9].

In the present study we investigated HUSK-participants' (1997-99) use of prescription CVM in 2008 (record linkage with NorPD data). CVMs were defined as drugs with ATC codes C01 (cardiac therapy), C02 (antihypertensives), C03 (diuretics), C07 (beta blocking agents), C08 (calcium channel blockers), C09 (agents acting on the renin-angiotensin system), and C10 (lipid modifying agents).

### Risk algorithms

On the basis of data recorded in HUSK during 1997-99, we were able to apply the following risk algorithms, which are all intended for risk stratification for primary prevention of CVD:

• The Framingham risk algorithm published in 1991 predicts the ten year risk for all kinds of coronary events, fatal and non-fatal [1]. A Framingham risk score of ≥20% is regarded as high risk.

• The SCORE (Systematic Coronary Risk Evaluation) risk algorithm presented in 2003 is based on a pooled dataset of cohort studies from 12 European countries, including Norway [2]. It predicts any kind of fatal CVD event over a ten-year period. The threshold for being at high risk is defined as ≥5%.

We extrapolated the values of Framingham and SCORE to the age of 60 years.

• The NORRISK risk algorithm [3]. This risk model from 2008 is based on updated Norwegian data regarding age- and sex specific CVD mortality rates. The NORRISK algorithm estimates the ten year risk of fatal CVD by combining gender, age, systolic blood pressure, total cholesterol and smoking. The threshold for high risk is set at ≥5%.

### Statistics and ethics

Statistical analyses were carried out using SPSS version 18. Bivariate comparisons were done by chi-square test (categorical variables) or independent samples t-test (conituous variables).

The study was approved by the Regional Committee for Medical Research Ethics and the Norwegian Data Inspectorate, for the 1997-99 as well as the 2008 data collection. A written consent allowing future use of their data for research was obtained from each HUSK-participant at the screening station.

## Results

During the 9-11 years from 1997-1999 to 2008, 26% of men (n = 2 411) and 22% of women (n = 2 448) not on CVM in 1997-99, had started such treatment (Table 2). 14.4% of men and 9.4% of women used more than one CVM. 14.2% of men and 9.6% of women used a statin and 5.6% of men and 4.6% of women used a statin as their only treatment. The proportion using CVM tended to increase with patients' age: for men from 21.3% for those born in 1957 to 34.1% for those born in 1950, corresponding figures for females were 17.1% and 30.4% (Table 2).

**Table 2:** Use of cardiovascular medication (CVM) in 2008 among persons not on treatment in 1997-99: Males: n = 9 283, females: n = 10 969

*Birth* *year Sex*		*CVM (%)*			
	
		*CVM*	≥*1 drug*	*Statin*	*Statin alone*
1950	M	34.1	20.0	18.8	7.1
	F	30.4	11.5	13.4	7.1

1951	M	28.7	15.8	16.6	6.4
	F	24.9	10.4	10.9	5.8

1953	M	30.0	17.2	16.0	6.2
	F	23.5	10.9	10.5	4.6

1954	M	26.9	15.5	15.3	5.4
	F	24.8	10.7	11.4	6.1

1955	M	23.4	12.6	12.6	5.1
	F	20.6	8.0	9.1	4.5

1956	M	22.5	12.3	12.0	5.1
	F	19.7	8.6	7.6	3.3

1957	M	21.3	10.7	11.5	5.4
	F	17.1	7.1	6.5	2.9

***Total***	M	26.0	14.4	14.2	5.6
	F	22.3	9.4	9.6	4.6

Table 3 shows the 1997-99-values for the Framingham, SCORE and NORRISK cardiovascular risk algorithms for those who had started medication during the period from 1997-1999 to 2008 versus those who had not. For both genders all risk scores showed significantly lower values for those not on CVM compared to those now on treatment. Female CVM users had significantly lower total risk score values compared to both males taking and not taking CVM (p-values not shown in table).

**Table 3:** Cardiovascular risk scores assessed in 1997-1999 for males (n = 2 411) and females (n = 2 448) using cardiovascular medication (CVM) in 2008 versus those not on CVM (males: n = 6 872, females: n = 8 521)^1^.

	*Males*			*Females*		
***Risk score algorithm***	***CVM***	***No CVM***	***95% CI for difference***	***CVM***	***No CVM***	***95% CI for difference***

Framingham	13.1	8.8*	3.9,4.5	5.7	3.2*	2.3,2.6

Framingham at 60 y	25.6	19.1*	6.0,6.9	12.7	8.1*	4.2,4.8

SCORE	1.8	1.2*	0.59,0.76	0.2	0.1*	0.09,0.10

SCORE at 60y	8.2	5.7*	2.3,2.7	2.1	1.4*	0.69,0.78

NORRISK	1.20	0.78*	0.36,0.47	0.32	0.20*	0.11,0.13

^1^Means with 95% CI for the difference

*p < 0.001 for difference in mean risk scores for males/females on CVM, versus males/females not on CVM. Independent samples t-test

Table 4 shows the proportion of men and women with cardiovascular risk scores at threshold or above and below "high risk" values in 1997-99 who used CVM in 2008. For this calculation we chose a "high risk" limit for NORRISK at ≥1% [3], as only 13 males and no females in our study population scored equal to or above the somewhat arbitrarily set limit of 5%. Among men with risk scores below the critical limits for the three different risk algorithms, 16-20% were using some CMV in 2008, for women this proportion was 20-22%. Among men with risk score values on thershold or above the "high risk" limits, 35-40% used CVM in 2008. Among the relatively few women above these limits, 56-72% had started using some CVM. Among women using CVM, only 2.2% (NORRISK), 4.4% (SCORE) and 14.5% (Framingham) had risk scores above the high-risk thresholds. The table also shows that 83% of men and 88% of women with blood pressure above 150/90 were treated, while 58% of men and 64% of women with a cholesterol level higher than 8 mmol/l used CVM.

**Table 4:** Proportions (numbers) of males and females using cardiovascular medication (CVM) in 2008 by their cardiovascular risk scores in 1997-1999.

*Risk score*	*Males (n = 9 283)*	*Females (n = 10 969)*
	
	*Use of CVM (%)*	
Framingham 60y ≥ 20%	37.1 (n = 1 533)	56.2 (n = 356)

Framingham 60y < 20%	17.1 (n = 878)	20.2 (n = 2 092)

SCORE 60y ≥ 5%	34.6 (n = 1 712)	76.1 (n = 108)

SCORE 60y < 5%	16.1 (n = 699)	21.6 (n = 2 340)

NORRISK ≥1%	40.0 (n = 1 071)	72.6 (n = 53)

NORRISK < 1%	20.3 (n = 1 340)	21.9 (n = 2 395)

BP > 150/95 mm Hg	82.7 (n = 7 677)	87.9 (n = 9 642)

Cholesterol > 8 mmol/l	58.1 (n = 5 393)	64.4 (n = 7 064)

Table 5 shows characteristics for females and males taking or not taking CVM. Compared to women not using CVM, women on medication reported poorer general and mental health, more muscular pains, more use of other medications, less physical exercise, less use of alcohol, and more were smokers. They also had a lower educational level, and reported a higher frequency of CVD in close relatives. Their BMI, SBP, DBP, total cholestrol, triglycerides, and glucosis values were all significantly higher and their HDL-cholesterol lower compared to those not using CVM. For men, there was no difference regarding mental health, use of alcohol or smoking between those using or not using CVM. For the remaining factors, the differences between the groups corresponded to those seen among females.

**Table 5:** Characteristics for females (n = 2 448) and males (n = 2 411) started on cardiovascular medication (CVM) versus those not on treatment (females n = 8 521, males n = 6 872) in the period 1997-2008^1^.

Characteristics 1997-99	Females			*Males*		
	
	CVM (n = 2 488)	No CVM (n = 8 521)	p-value*	*CVM (n = 2 411)*	*No CVM (n = 6 872)*	p-value*
General health less than good	22.9	12.7	<0.001	16.1	10.7	<0.001

General health very good	14.1	23.5	<0.001	13.4	20.9	<0.001

Anxiety last 2 weeks^1^	4.2	2.5	<0.001	2.4	2.0	0.2

Depression last 2 weeks^1^	6.6	5.2	0.004	4.0	3.5	0.3

Muscular pain ≥ 3 months	56.0	45.9	<0.001	40.8	37.3	0.002

Use of other medication	63.7	50.4	<0.001	43.6	28.3	<0.001

Hard exercise < 1 hr/week	60.0	56.5	0.002	55.5	51.3	<0.001

Light exercise < 1 hr/week	18.3	15.0	<0.001	22.8	20.2	0.007

No use of alcohol	11.5	9.7	0.009	6.7	7.2	0.4

Daily cigarette smoking	38.0	35.8	0.04	36.5	34.5	0.07

Education < 12 years	61.8	52.8	<0.001	56.3	51.4	<0.001

Education ≥ 16 years	11.9	17.0	<0.001	15.0	18.9	<0.001

MI in relatives < 60 years	22.7	14.9	<0.001	23.0	13.7	<0.001

Stroke in relatives < 70 years	14.9	10.3	<0.001	13.0	9.2	<0.001

BMI, kg/m^2^, mean (SD)	26.5 (4.8)	24.3 (3.7)	<0.001	27.4 (3.6)	25.7 (3.6)	<0.001

SBP, mmHg, mean (SD)	132.7 (17.2)	120.8 (12.2)	<0.001	138.7 (15.3)	128.7 (15.3)	<0.001

DBP, mmHg, mean (SD)	76.9 (11.4)	69.3 (9.0)	<0.001	82.6 (11.0)	74.7 (8.9)	<0.001

Chol, mmol/l, mean (SD)	5.8 (1.1)	5.3 (0.9)	<0.001	6.1 (1.3)	5.6 (1.0)	<0.001

Hdl chol, mmol/l, mean (SD)	1.31 (0.38)	1.42 (0.33)	<0.001	1.06 (0.26)	1.15 (0.29)	<0.001

TG, mmol/l, mean (SD)	1.72 (1.16)	1.29 (0.73)	<0.001	2.62 (2.17)	1.99 (1.20)	<0.001

Glucosis, mmol/l, mean (SD)	5.3 (1.4)	5.0 (0.9)	<0.001	5.5 (1.3)	5.2 (1.0)	<0.001

^1^Figures are proportions unless otherwise stated

Myocardial infarction (MI), Body mass Index (BMI) Standard deviation (SD)

Systolic Blood Pressure (SBT); Diastolic Blood Pressure (DBT); High Density Lipid (HDL); TriGlycerides (TG)

^1^Substantially or very much bothered

*p-value for difference in propotions or in mean levels of each characteristic for females/males using CVM versus females/males not using CVM (chi square test/independentsamples t-test)

Compared to men on CVM, women on treatment reported poorer general and mental health, more muscular pains, more use of other medications, and fewer carried out hard physical exercise (Table 5, p-values not shown in Table). However, relatively more men reported they were physically inactive, and fewer reported no use of alcohol. Women had more favourable values for BMI, SBP, DBP, total- and HDL-cholestrol, triglycerides and glucosis compared to men, and they were generally less educated compared to their male counterparts.

For both genders, all risk algorithms showed a significant correlation with mental distress (Table 6).

**Table 6:** Mean CVD risk scores (SD) related to mental distress

*Risk score*	*Males*				*Females)*			
	
	*Anxiety*		*Depression*		*Anxiety*		*Depression*	
	
	*Yes* *n = 186*	*No* *n = 8618*	*Yes* *n = 317*	*No* *n = 8492*	*Yes* *n = 293*	*No* *n = 9908*	*Yes* *n = 561*	*No* *n = 9966*
*Framingham*	12.1 (6.9)	9.9* (6.1)	11.1 (6.5)	9.9* (6.2)	5.1 (4.0)	3.7* (3.5)	4.5 (3.8)	3.7* (3.5)

*SCORE*	1.67 (1.16)	1.33* (1.87)	1.54 (1.12)	1.33* (1.88)	0.20 (0.16)	0.16* (0.15)	0.18 (0.17)	0.16* (0.15)

*NORRISK*	1.08 (0.69)	0.87* (0.61)	1.00 (0.65)	0.88* (0.61)	0.27 (0.20)	0.23* (0.18)	0.25 (0.19)	0.23* (0.18)

*p < 0.01 (independent samples t-test) for difference in risk score by categories of self reported mental distress:

Yes = substantially or very much bothered

No = not or a little bothered

Out of the 22 241 persons originally included in the study, 343 persons were reported to have died at some point between the data collection in 1997-99 and December 2009 (data from the National Population Registry). 152 out of these 343 persons could be traced in the Norwegian Cancer Registry [7]. Among the remaining 191 individuals, some probably died of cardiovascular disease, but we do not consider this small number to influence our results significantly.

## Discussion

In this study we found that close to one in four persons 51-58 years of age had started to use some CVM during the previous decade: 22% of women and 26% of men. Around one in eight used more than one drug and a corresponding proportion used a statin.

High cardiovascular risk scores (Framingham, SCORE and NORRISK) calculated 9-11 years previously were all significantly associated with subsequent medication use, although there was a substantial overlap: A considerable proportion of individuals with risk scores below the "high-risk" thresholds used CVM (males from 16 to 20% of the cohort for the three included risk algorithms, females 20-22%). Among women using CVM, at least eight out of ten had risk score values below the defined "high-risk" thresholds. On the other hand, around 60% of men with risk scores above the "high risk" level, did not use any CVM. Even in the group of men with risk score levels on or above the 90^th ^percentile (corresponding to Framingham at 60 years 33.87, SCORE at 60 years 11.15 and NORRISK 1.61) 47-48% used no CVM (data not shown in table). It may thus seem that prescription of CVM to a large degree had occured independently of the patient's total cardiovascular risk, as expressed by these commonly used risk algorithms.

Maybe even more striking is that the proportion of females on medication roughly corresponded to that of males (22.3% versus 26.0%), despite females' substantially lower risk score levels-the majority of women on CVM had risk score levels well below the "high-risk" limits.

The study has some limitations. It was done by a population survey and a subsequent linkage to a prescription database, not by intentionally following a well designed cohort. Among others, we can not tell if the medications had been prescribed for present CVD or for primary- or secondary prevention. The response rate of the population survey was as high as 66.6%, but those not participating were found to differ from participants on several aspects, as non-participants were less educated, had lower income and more frequently received social security grants [7]. There is reason to believe that their risk profiles also differ from the respondents' [10]. Furthermore, the results presented in Table 5 should be interpreted with caution, due to multiple testing. The strengths of the study were the high response rate in HUSK, the high reliability of the measurements and analyses carried out at the mobile screening stations, as well as the subsequent linkage to a prescription database comprising all prescriptions in Norway, securing a close to complete follow-up.

The cohort of women on CVM differed from those not on medication regarding several parameters: They had generally lower education, their self-reported health was poorer, and they more frequently reported muscular pains, anxiety and depression. They also smoked more, exercised less, and more frequently abstained from alcohol. Compared to men on CVM, women on treatment reported poorer general health, more muscular pains, anxiety, and depression. Psychosocial factors have been extensively linked to CVD [10-12]. In a systematic review of prospective cohort studies of healthy populations Hemingway and Marmot highlighted a probable role for among others anxiety, depression and social support in the aetiology and prognosis of coronary heart disease [10]. In a Dutch study, anxiety was found to predict cardiovascular death during a 10-year follow-up among middle-aged women (hazard ratio 2.77, 95% CI 1.17,6.85) [11], while in a study from 52 countries, the presence of psychosocial stess was found to be associated with increased risk of myocardial infarction [12]. In our study, anxiety and depression correlated significantly with risk score levels for both genders, but correlated with CVM use only in females, while a low educational level corrrelated with use of CVM in both genders. In a recent Finnish study an association was found between psychological distress and cardiovascular risk scores (Framingham and SCORE) for men, but not for women [13]. Our results may reflect that clinicians, in particular for female patients, tend to include features like mental distress and social situation when deciding if and when a patient needs CVM [14].

In our study population covering a possible age span from 40 to 58 years, more than one in four males and one in five females had started on CVM during a decade. Recommended risk algorithms for CVD at baseline only to a limited degree correlated with later medication use, especially in females, while psychosocial factors correlated stronger with use of CVM in females compared to males. Possible reasons for underusing total risk assessment tools in clinical practice may be that the tools are considered inaccurate or poorly validated. Whether their use improves patient outcomes has in fact rarely been evaluated [15-17]. Furthermore, critics have emerged that guidelines for preventive cardiology (which form the basis for the application of the risk assessment tools) tend to overestimate CVD risk and hence target a too large share of the population for risk interventions [18,19]. The Framingham equations have been found to over-predict coronary events by 57% compared with observed events in a representative sample of British men [20]. NORRISK is more adapted to the current Norwegian risk situation compared to the SCORE and Framingham algorithms, both of which have been found to overestimate the risk of fatal CVD in Norway [21]. However, our study reveals that none of the three risk algoritms proved to be superior to the others in order to predict subsequent use of CVM in a middle aged cohort. Most females on medication had risk score values well below risk-thresholds, while less than half of men with values above the threshold limits used CVM.

The newly developed CVD risk score QRISK2 includes the traditional Framingham risk factors, but also includes BMI, a family history of CVD, social class measured by Townsend score, self-assigned ethnicity, and presence of several listed diseases known to be associated with CVD, among others diabetes mellitus and rheumatoid arthritis [4]. This score has been externally validated, and is found to predict cardiovascular risk more accurately than the Framingham score [22].

Official guidelines on calculating global risk were implemented in Norway in 2009, that is after our data collection took place, but GPs had already been educated in global risk assessment during the previous 20 years, through guidelines provided by the National College of GPs. We would therefore expect the average physician to be informed about the recommendations regarding total risk assessment in 2008. But even if clinicians do calculate patients' total risk score, little is known about how this knowledge affects the physicians' decision making in terms of prescribing CVM [17]. We have not found any earlier study linking use of CVM directly to CVD-risk in a selected population cohort. The results of our study indicate that clinicians still probably tend to base their prescription decisions on other features than a total CVD risk assessment: a family history of CVD, single risk-factor assessment (elevated BP- or lipid values), or psychosocial factors. In our study, the strongest correlation between one single measure and CVM was found for BP. With a BP > 150/95, as many as 83% of men and 88% of women received drug treatment. However, we still have limited knowledge on how clinicians decide which patient to put on treatment, and when. Such knowledge is important to ensure that the relatively large population share on CVM includes those individuals who will benefit most from using them, probably for the rest of their lives.

## Conclusion

Prescription of cardiovascular medication to middle-aged persons to a large degree was found to occur independently of the indviduals' total cardiovascular risk, especially in women. Despite their substantially lower total cardiovascular risk, middle aged women by large recieved as much cardiovascular medications as men. At least eight out of ten middle aged women using cardiovascular medication in 2008 had cardiovascular risk scores (Framingham, SCORE, NORRISK) below defined "high-risk" thresholds ten years before.

Cardiovascular drug prescription practice still seems to be based on single factor assessments (blood pressure, lipid level, family history of CVD), but psychosocial factors seem to influence which patients will be put on treatment, especially in women.

## Competing interests

The authors declare that they have no competing interests.

## Authors' contributions

The authors planned the study together. MB carried out the data analyzes and drafted the article. JS actively participated in revising the manuscript. Both authors read and approved the final manuscript.

## Pre-publication history

The pre-publication history for this paper can be accessed here:

http://www.biomedcentral.com/1471-2458/11/144/prepub
